# 4-Ethylphenol, A Volatile Organic Compound Produced by Disease-Resistant Soybean, Is a Potential Botanical Agrochemical Against Oomycetes

**DOI:** 10.3389/fpls.2021.717258

**Published:** 2021-09-22

**Authors:** Ting Ge, Wenteng Gao, Changhui Liang, Chao Han, Yong Wang, Qian Xu, Qunqing Wang

**Affiliations:** ^1^Shandong Province Key Laboratory of Agricultural Microbiology, Department of Plant Pathology, College of Plant Protection, Shandong Agricultural University, Tai’an, China; ^2^State Key Laboratory of Crop Biology, Shandong Agricultural University, Tai’an, China; ^3^Shimadzu (China) Co., Ltd., Beijing, China; ^4^College of Agronomy, Shandong Agricultural University, Tai’an, China

**Keywords:** 4-Ethylphenol, leaf volatile compounds, cell membrane damage, biological control, *Phytophthora*

## Abstract

Oomycetes, represented by *Phytophthora*, are seriously harmful to agricultural production, resulting in a decline in grain quality and agricultural products and causing great economic losses. Integrated management of oomycete diseases is becoming more challenging, and plant derivatives represent effective alternatives to synthetic chemicals as novel crop protection solutions. Biologically active secondary metabolites are rapidly synthesized and released by plants in response to biotic stress caused by herbivores or insects, as well as pathogens. In this study, we identified groups of volatile organic compounds (VOCs) from soybean plants inoculated with *Phytophthora sojae*, the causal agent of soybean root rot. 4-Ethylphenol was present among the identified VOCs and was induced in the incompatible interaction between the plants and the pathogen. 4-Ethylphenol inhibited the growth of *P. sojae* and *Phytophthora nicotianae* and had toxicity to sporangia formation and zoospore germination by destroying the pathogen cell membrane; it had a good control effect on soybean root rot and tobacco black shank in the safe concentration range. Furthermore, 4-Ethylphenol had a potent antifungal activity against three soil-borne phytopathogenic fungi, *Rhizoctonia solani*, *Fusarium graminearum*, and *Gaeumannomyces graminis* var *tritici*, and four forma specialis of *Fusarium oxysporum*, which suggest a potential to be an eco-friendly biological control agent.

## Introduction

Oomycetes, encompassing *Phytophthora*, *Albugo*, *Pythium*, and a group of downy mildews that cause plant epidemics, have a negative impact on natural and farm ecosystems due to their strong pathogenicity and infectivity (Yutin et al., 2008; Kamoun et al., 2015). Besides the well-known potato late blight caused by *Phytophthora infestans*, which led to the Irish famine of the 19th Century, *Phytophthora nicotianae* is a pathogen distributed worldwide, and it causes tobacco black shank and is responsible for many foliar and fruit diseases (Fang et al., 2016). Soybean root rot is caused by *Phytophthora sojae* and is the leading cause of global soybean production losses (Tyler, 2007). Oomycetes are phylogenetically different from fungi, forming an independent group, and are therefore resistant to many broad-spectrum fungicides (Tyler et al., 2006). Some of the fungicides effective against oomycetes, such as metalaxil, have resulted in the emergence of insensitive strains and resurgence events due to their single site of action (Randall et al., 2014). Interdisciplinary studies and consistent resources have been invested in finding new, effective alternatives for the integrated pest management of oomycete diseases (Gessler et al., 2011).

Novel pharmaceuticals against oomycetes should be explored for rational fungicide design, and further focus should be placed on developing alternative botanical agrochemicals to fight different pathogens that attack crops and related products (Drakopoulos et al., 2020; Liang et al., 2021; Wang et al., 2021). Environmentally friendly botanical fungicides are widely welcomed due to their higher efficiency, lower residue, and lower negative impact on the environment (Naz et al., 2018; Tschoeke et al., 2019). Plants are a rich natural source of active antimicrobial substances (Nino et al., 2012; Hu et al., 2018). For example, artemisinin, present in sweet wormwood, a Chinese medicinal plant, is the most effective antimalarial drug available (Tu, 2011).

Natural products have a long history as a source of novel agrochemicals (Yoon et al., 2013). Phytopathologists search for alternative botanical products to replace synthetic fungicides and effectively control plant diseases without significantly affecting crop yields (Bowers and Locke, 2000). For example, poacic acid, which is derived from grass lignocellulosic hydrolysates, inhibits the growth of the *Sclerotinia sclerotiorum* and *Alternaria solani* fungi and the oomycete *P. sojae* (Piotrowski et al., 2015). Pathogen cells treated with poacic acid suffer similar effects to those treated with cell wall-targeting synthetic drugs (Lee et al., 2018). There is considerable evidence for the protective effects of phytochemicals isolated from tissue exudates or volatiles against disease propagation (Drakopoulos et al., 2020; Liao et al., 2021; Wang et al., 2021). For example, grape cane ε-viniferin has antifungal activity against *Plasmopara viticola* and *Botrytis cinerea* (Schnee et al., 2013). Secomicromelin, coumarin, isomicromelin, and micromarin B, present in *Micromelum falcatum* fruits, inhibit the growth of *Pythium insidiosum* (Suthiwong et al., 2014). Cuminic acid, isolated from cumin seeds (*Cuminum cyminum* L.), inhibits *Phytophthora capsici* mycelial growth and zoospore germination (Wang et al., 2016). Gossypol, naturally present in cotton root tissues, has a strong inhibitory activity on *Pythium irregulare*, *Pythium ultimum*, and *Fusarium oxysporum* growth (Mellon et al., 2014).

Leaf volatile organic compounds (VOCs) are rapidly emitted when plants respond to biotic stress caused by herbivores or attacks by necrotrophic fungi (Scala et al., 2013; Matsui and Koeduka, 2016; Tanaka et al., 2018). VOC production is a basic defense mechanism for plants to enhance resistance or tolerance to upcoming stresses and may contribute to direct plant defense responses through their powerful antimicrobial activities (Jerkovic et al., 2012; Krajaejun et al., 2012; Ulloa-Benitez et al., 2016; Ricciardi et al., 2021).

Here, we analyzed soybean leaf volatiles produced in incompatible interaction and compatible interaction with *P. sojae*. A group of VOCs was identified by headspace solid-phase microextraction coupled with gas chromatography–mass spectrometry (HS–SPME–GC–MS), which were specifically present in the incompatible interaction. In Petri dish assays, 4-Ethylphenol, a volatile phenolic substance, inhibited the mycelial growth, sporangia formation, and zoospore germination of *P. sojae* and *P*. *nicotianae*. Additionally, it had potent antifungal activities against three soil-borne phytopathogenic fungi, *Rhizoctonia solani*, *Fusarium graminearum*, *Gaeumannomyces graminisvar*, and four *Fusarium oxysporum* forma specialis. We found that 4-Ethylphenol triggers mycelia malformation and cytoplasmic electrolyte leakage because of a disrupted or disintegrated plasma membrane. Finally, we analyzed the potential of 4-Ethylphenol as an oomycete biological control agent and confirmed its efficacy in controlling soybean root rot and tobacco black shank diseases in potted plants, and observed a positive effect on plant growth when present in low concentrations.

## Materials and Methods

### Plant and *Phytophthora* spp. Cultivation

Soybean and tobacco plants were grown in a chamber at 25°C, with a cycle of 16 h of high light intensity and 8 h of darkness. *P. sojae* strain P6497 and *P. nicotianae* strain INRA-310 were grown on 10% V8 medium (10% V8 juice, 0.02% CaCO_3_, and 1.5% agar) in the darkness at 25°C. Mycelia were cultured in V8 liquid medium. To observe zoosporangia, the mycelia were washed with sterile water five times and cultured in the darkness at 25°C for 6 h. When zoosporangia formed, zoospores were released after washing with sterile water at 10°C three times. Finally, the concentration of the zoospore suspension was adjusted to 10^5^ CFU/mL.

### Gas Chromatography–Mass Spectrometry Analysis

Soybean leaves (Williams and Williams-82 cultivars) inoculated with *P. sojae* were placed in a 20 mL headspace bottle, and the VOCs were analyzed with gas chromatography–mass spectrometry (GC–MS). An AOC-6000 Multifunctional Autosampler was used for solid-phase microextraction injection, and GCMS-TQ8040 NX was used for detection following the standard SPME parameters (SPME fiber: FIB-C-WR-95/10. The following parameters were used: aging temperature, 240°C; aging time (before extraction), 30 min; equilibration temperature, 40°C; equilibration time, 5 min; extraction time, 30 min; injection port temperature, 250°C; desorption time, 2 min; and aging time (after extraction), 5 min. The GC–MS/MS parameters used were: column, inert cap pure-wax, 30 m × 0.25 mm × 0.25 m; oven program, 50°C (5 min), 10°C/min_250°C (10 min); carrier gas pressure, 83.5 kPa; injection mode, split; split ratio, 5:1; ion-source temperature, 200°C; interface temperature, 250°C; detector voltage, tuning voltage + 0.3 kV; and acquisition mode, MRM.

### Effect of 4-Ethylphenol on the Radial Growth of *Phytophthora* spp. Hyphae

Hyphal plugs of *P. sojae* and *P. nicotianae* were cultured in 10% V8 agra medium containing different concentrations of 4-Ethylphenol, or the same volume of sterile water as a control. The medium was incubated in the darkness at 25°C for 5 days; then, the colony diameter was measured, and the mycelium status was observed under the microscope. Each treatment was repeated three times.

### Effects of 4-Ethylphenol on *Phytophthora* spp. Zoosporangium Formation and Zoospore Release

Washed mycelia were placed in different concentrations of 4-Ethylphenol. After incubation at 25°C for 6 h, the number of zoosporangia was observed and recorded under the microscope using Mallassez cell counting. 4-Ethylphenol was added to the sporangium-forming dishes, and after 2 h, the number of zoospores was measured under the microscope using Mallassez cell counting. Each treatment was repeated three times.

### Effects of 4-Ethylphenol on *Phytophthora* spp. Zoospore Germination

The 0.1 mL zoospore suspension was evenly spread on V8 medium containing four different concentrations of 4-Ethylphenol. After incubation at 25°C for 4 days, the minimum concentration with no colony formation was determined by naked eye observation. Each treatment was repeated three times.

### Effect of 4-Ethylphenol on *Phytophthora sojae* Virulence

Soybeans were planted in the dark for 7 days, and etiolated seedlings were immersed in 4-Ethylphenol solution for 1 h and then placed in zoospore suspension for infection. After 4 or 6 h of infection, the hypocotyls were collected and stained with a lactophenol–trypan blue dye solution. After 2 h of staining, samples were destained with chloral hydrate until they were translucent. The discolored epidermis was then removed with forceps, prepared, and observed under the microscope. Each treatment was repeated three times.

### Damage of the *Phytophthora* spp. Cell Membrane by 4-Ethylphenol

Mycelia were cultured in V8 liquid medium for 3 days. Washed mycelia were placed in PBS buffer (pH 7.0) containing 4-Ethylphenol; DNA and protein concentrations were measured and recorded every 2 h. Each treatment was repeated three times.

### Safety of 4-Ethylphenol on Soybean and Tobacco Plants

Different concentrations of 4-Ethylphenol (0–25 mg a.i./plant) were mixed with soil. The growth and development of soybean and tobacco plants were recorded at 7 and 14 days after treatment, respectively. Each treatment was repeated three times.

### Efficacy of 4-Ethylphenol as a Soil Fumigant

Different concentrations of 4-Ethylphenol were mixed with soil and then sealed in plastic film for 15 days. After being air-cured for 2 days, soybean and tobacco were planted in the soil, and their growth and development were observed after 9 days. Each treatment was repeated three times.

### Statistical Analysis

Statistical analysis was performed using Statistical Product and Service Solutions (SPSS) 19.0. The difference among treatments was determined based on one-way analysis of variance (ANOVA), and means were subjected to Duncan’s multiple range test with significance set at *P* < 0.05.

## Results

### Detection of the VOC 4-Ethylphenol in Soybean Leaves

We investigated the VOCs produced in the leaves of susceptible soybean plants (Williams), lacking resistance genes to *P. sojae* (*Rps*), and resistant soybean plants (Williams82), containing the *Rps1k* resistance gene, by GC—–MS. The 4-Ethylphenol content in Williams82 was significantly higher than that in Williams leaves (Supplementary Figure 1), suggesting that 4-Ethylphenol is involved in the defense response of soybean to *P. sojae*.

### Effect of 4-Ethylphenol on the Mycelium Growth of *Phytophthora* spp.

**FIGURE 1:**
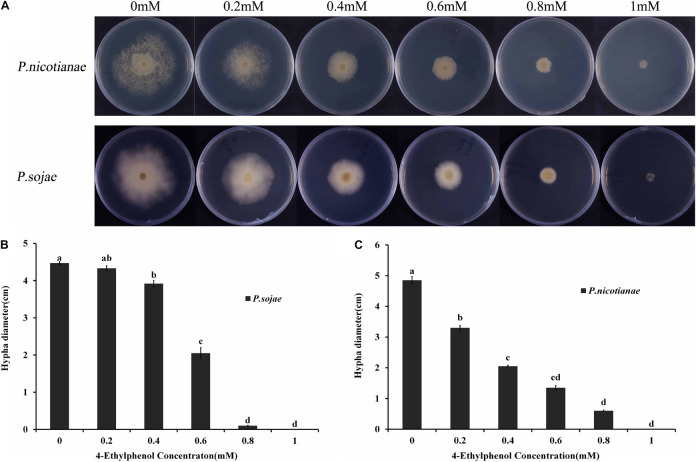
Antifungal activity of 4-Ethylphenol against *Phytophthora* spp. **(A)** *P. sojae* and *P. nicotianae* were cultured on V8 medium with different concentrations of 4-Ethylphenol. **(B)** Colony diameters of *P. sojae* were measured after 5 days. **(C)** Colony diameters of *P. nicotianae* were measured after 5 days. The experiment was repeated three times with similar results.

The radial diameter of *P. sojae* and *P. nicotianae* colonies grown on V8 medium supplemented with different concentrations of 4-Ethylphenol was determined through naked eye observation; the toxicity of 4-Ethylphenol to *Phytophthora* spp. was observed and calculated (Figure 1A). The average diameter of *P. nicotianae* colonies cultured for 5 days on V8 medium containing 0.4 mmol (57.66 mg/L) of 4-Ethylphenol was 20.52 mm, and the inhibition rate was 57.73% (Figure 1B). Additionally, the average diameter of *P. sojae* colonies cultured for 5 days on V8 medium containing 0.6 mmol (86.48 mg/L) of 4-Ethylphenol was 20.50 mm, and the antifungal rate was 54.14%. Medium containing 1 mmol (144.14 mg/L) of 4-Ethylphenol completely inhibited the growth of both oomycete species (Figure 1C).

### Effect of 4-Ethylphenol on the Morphology and Cell Membrane of *Phytophthora* spp. Hyphae

**FIGURE 2:**
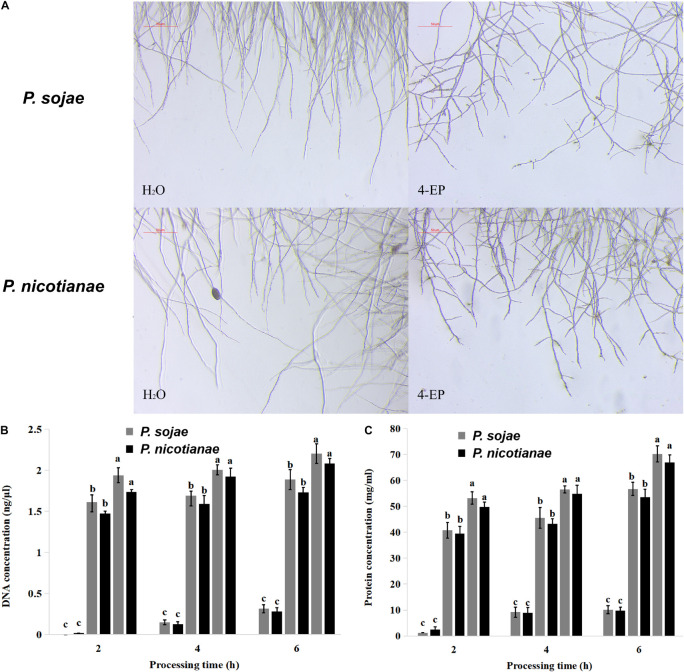
Effect of 4-Ethylphenol on *Phytophthora* spp. morphology and DNA and protein leakage. **(A)** Effect of 4-Ethylphenol on the morphology of *P*. *sojae* and *P. nicotianae* treated with 4-Ethylphenol for 2 h; **(B)** effect of 4-Ethylphenol on DNA leakage of *P. sojae* and *P. nicotianae*; **(C)** effect of 4-Ethylphenol on protein leakage of *P. sojae* and *P. nicotianae*.

*Phytophthora* spp. hyphae grew naturally in V8 medium, without increased terminal branching; the mycelium growing points were uniform, and the branches formed far from the top. After treatment with 0.4 mmol of 4-Ethylphenol, the growth of *P. sojae* and *P. nicotianae* hyphae was inhibited in the V8 liquid medium. The morphology of the mycelia changed in the presence of 4-Ethylphenol, and the branches at the end increased significantly and became disordered (Figure 2A). The results showed that 4-Ethylphenol inhibited growth by changing the morphology of *Phytophthora* spp. hyphae. To analyze the mechanisms of antimicrobial activity against *P. sojae* and *P. nicotianae*, we treated mycelia with 4-Ethylphenol and investigated the presence of leakage of cellular material. By monitoring the total DNA and protein concentrations in the media every 2 h, we observed that the contents increased significantly with time, and the intensity of leakage increased with 4-Ethylphenol concentration (Figures 2B,C). The leakage of cellular contents was likely due to the destruction of the cell membrane by 4-Ethylphenol.

### Inhibition Effect of 4-Ethylphenol on the Formation of Sporangium and Zoospores of *Phytophthora* spp.

**FIGURE 3:**
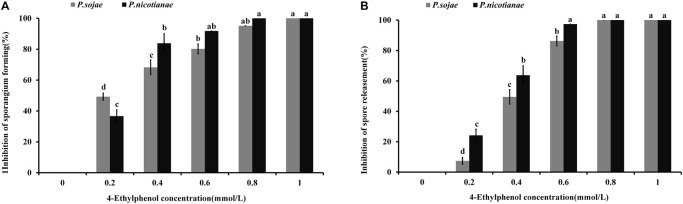
Effect of 4-Ethylphenol on zoosporangium formation and zoospore release. **(A)** Inhibition rate of 4-Ethylphenol on *P. sojae* and *P. nicotianae* zoosporangium formation; **(B)** inhibition rate of 4-Ethylphenol on *P. sojae* and *P. nicotianae* zoospore release. The experiment was repeated three times with similar results.

To study the effect of 4-Ethylphenol on *Phytophthora* spp. zoosporangia formation, we determined the number of zoosporangia and calculated the inhibition degree after treatments with different concentrations of the VOC. The formation of *P. sojae* zoosporangia was significantly reduced by 0.2 mmol of 4-Ethylphenol, while no zoosporangium was formed with 1 mmol. For *P. nicotianae*, 0.4 mmol of 4-Ethylphenol significantly reduced zoosporangium formation, whereas 0.8 mmol of 4-Ethylphenol completely inhibited it (Figure 3A).

Additionally, zoospore release from both *P. sojae* and *P. nicotianae* sporangia was significantly reduced with 0.4 mmol of 4-Ethylphenol, and completely inhibited with 0.8 mmol of 4-Ethylphenol (Figure 3B).

### Effect of 4-Ethylphenol on *Phytophthora* spp. Zoospore Germination

To analyze the effect of 4-Ethylphenol on *Phytophthora* spp. zoospore germination, we spread a zoospore suspension evenly on 1% V8 medium containing different concentrations of the VOC. Compared with control, 0.4 mmol of 4-Ethylphenol significantly inhibited the germination of *P. sojae* zoospores; only a few zoospores could germinate and form separate colonies. At 0.8 mmol of 4-Ethylphenol, the germination of zoospores was completely inhibited. For *P. nicotianae*, 0.6 mmol of 4-Ethylphenol significantly inhibited zoospore germination, and 1 mmol completely inhibited it (Supplementary Table 1).

### Effect of 4-Ethylphenol on *Phytophthora sojae* Zoospore Invasion

**FIGURE 4:**
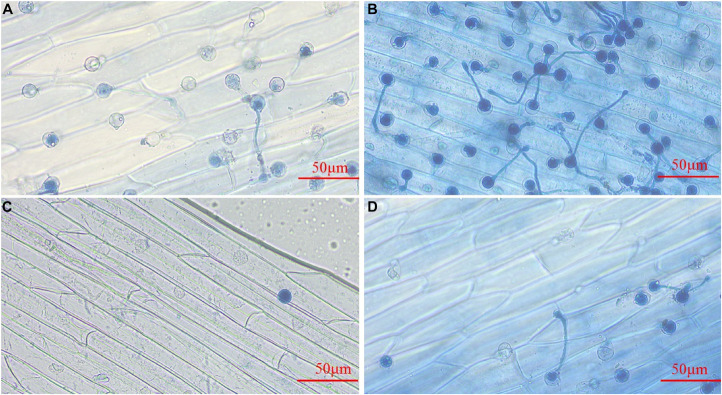
Effect of 4-Ethylphenol on *P. sojae* zoospore invasion. Zoospore invasion after **(A)** 4 h of the control treatment (water), **(B)** 6 h of the control treatment (water), **(C)** 4 h of the 4-Ethylphenol treatment, **(D)** 6 h of the 4-Ethylphenol treatment. The control-treated *P. sojae* zoospores attached to the hypocotyls of yellow–yellow seedlings and grew many hyphae for infection, whereas the *P. sojae* treated with 4-Ethylphenol significantly reduced the adhesion and infection ability. The experiment was repeated three times, and similar results were obtained.

*Phytophthora sojae* zoospores treated with the control could attach to the epidermis of soybean hypocotyls, and some of them could form germ tubes at 4 h post-inoculation (hpi) (Figure 4A). At 6 hpi, more zoospores were attached to the epidermis, and the majority had germinated hyphae for infection (Figure 4B). Treatment with 4-Ethylphenol significantly reduced the zoospore adhesion ability, and only a few attached to the surface of soybean hypocotyls at 4 hpi (Figure 4C). At 6 hpi, the zoospore attachment and germination decreased significantly compared with the control (Figure 4D), indicating that 4-Ethylphenol could effectively inhibit zoospores from infecting soybean.

### Effect of 4-Ethylphenol on Germination and Growth of Soybean and Tobacco Plants

**FIGURE 5:**
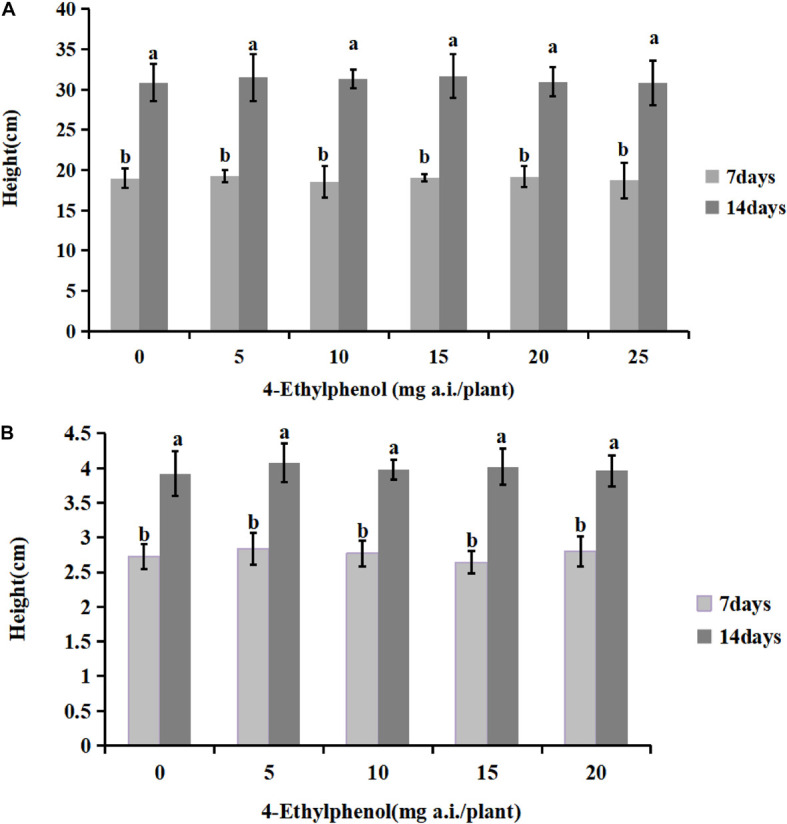
Effects of 4-Ethylphenol on soybean **(A)** and tobacco **(B)** height. Data are the mean ± S.E. from three replicates per treatment.

To investigate the safety of 4-Ethylphenol for host plants, we planted soybean and tobacco seedlings in soil mixed with different concentrations of 4-Ethylphenol, and seedling heights were measured 7 and 14 days after the treatment (Figure 5). The results showed that the germination rate of soybean plants under all tested concentrations reached 100%, indicating that 4-Ethylphenol did not affect soybean seed germination. The average height of soybean plants increased compared with the control with treatments at low concentrations, indicating that 4-Ethylphenol promoted the growth of soybean plants. The maximum concentration tested, 25 mg a.i./plant, had no obvious inhibitory effects on seedling height, indicating that 4-Ethylphenol did not affect the normal soybean growth (Figure 5A and Supplementary Figure 2A). Regarding tobacco plants, the average height of plants with 4-Ethylphenol was not significantly different from that of the control group after 7 and 14 days (Figure 5B and Supplementary Figure 2A). Tobacco seedlings treated with different concentrations grew normally and somewhat consistently.

### Efficacy of 4-Ethylphenol Against Soybean Root Rot and Tobacco Black Shank Diseases

**FIGURE 6:**
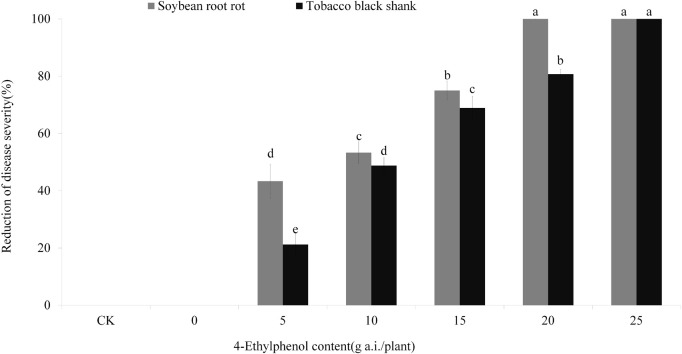
Efficacy of 4-Ethylphenol on soybean root rot and tobacco black shank diseases. Data are the mean ± S.E. from three replicates per treatment.

Based on the results from the concentration gradient safety test, we planted soybean and tobacco on soil mixed with 4-Ethylphenol and mycelia to observe the control effect on soybean root rot and tobacco black shank. The results showed that control-treated soybean plants (no pathogen) grew normally, whereas those treated with *P. sojae*-containing soil had serious disease symptoms (Figure 6 and Supplementary Figure 3B). With the increase in 4-Ethylphenol concentration, the disease index decreased gradually, and the relative control effect improved. When 20 mg a.i./plant of 4-Ethylphenol was mixed with soil, the control effect reached 100%, and soybean plants were healthy (Figure 6 and Supplementary Figure 3A). Furthermore, tobacco plants grown without the pathogen grew normally, whereas adding *P. nicotianae* to the soil prompt quick plant death. Application of 4-Ethylphenol decreased the disease index gradually in a dose-dependent response and the tobacco plants were mostly healthy. When 25 mg of 4-Ethylphenol was applied per pot, all tobacco plants grew normally, and the control effect reached 100% (Figure 6 and Supplementary Figure 3B).

## Discussion

Oomycetes are fungus-like organisms that include a group of notorious phytopathogens, namely *Phytophthora*, *Albugo*, *Pythium*, and downy mildews (Kamoun et al., 2015). Although oomycetes resemble filamentous fungi (Jiang and Tyler, 2012), they are phylogenetically related to diatoms and brown algae in the stramenopiles (Tyler et al., 2006; Haas et al., 2009; Thines and Kamoun, 2010; Tan et al., 2020). The well-documented Irish famine forced humans to investigate the causes of the potato late blight and discover the responsible microbial pathogen, *P. infestans* (Haas et al., 2009). Since the 1870s, breeding efforts for late blight resistance have failed to provide a durable, resistant cultivar (DeArce, 2008). To control late blight, farmers rely largely on fungicides with unknown modes of action (Rekanovic et al., 2012; Ferreira et al., 2014; Randall et al., 2014; Childers et al., 2015; Miao et al., 2016; Chen et al., 2018). Many fungicides are ineffective against oomycetes because of their phylogenetic differences (Siegenthaler and Hansen, 2021). Although some fungicides have had some effect on oomycetes, widespread use quickly caused fully insensitive races to emerge (Randall et al., 2014; Childers et al., 2015; Matson et al., 2015; Pang et al., 2016). Currently, *P. infestans* remains a major constraint to the global production of potato and tomato and is thus a constant threat to food security (Haverkort et al., 2008; Fisher et al., 2012). In addition to late blight, *Phytophthora* spp. soybean root rot and tobacco black shank are diseases that are distributed worldwide, which lack effective control methods (Dorrance et al., 2003; Cui et al., 2010; Ji et al., 2014; Li et al., 2017). Copper-based fungicides are effective in crop protection against oomycetes, but they are facing restrictions because of copper accumulation in the soil (Mackie et al., 2013; Wightwick et al., 2013; Thuerig et al., 2018). New sources of fungicides have to keep pace with the evolution of resistant strains and emerging pathogens (Alexander and Perfect, 1997; Jian et al., 2015).

Due to the increasingly stringent regulatory requirements, crop protection approaches will have to ensure environmental conservation and sustainability (Hao et al., 2019; Kapsi et al., 2019). The use of natural products as an alternative to synthetic chemicals in the fight against different phytopathogens remains a constant need (Thuerig et al., 2018; Lorsbach et al., 2019). The primary and secondary metabolites produced by plants are important sources for developing novel environmental-friendly agrochemicals (Dayan et al., 2009). VOCs synthesized and emitted in response to pathogen infection are particularly relevant and usually have functional benefits in multiple aspects of plant defense (Ricciardi et al., 2021; Xu et al., 2021).

In this study, we analyzed the VOCs emitted when soybean plants responded to *P. sojae.* We used an odor analyzer combined with a Triple Quadrupole quality selection detector GCMS-TQ8040NX (Simadzu Company, Kyoto, Japan) and AOC-6000 multifunctional automatic sampler to establish an analysis method for VOCs induced in plant leaves. Regarding the software, the Odor Analyzer provides a complete method package and database, which was first released and used to analyze plant VOCs. One hundred and fifty components were determined with this semi-quantitative method, which is easy to operate, rapid to provide and analyze data, and suitable for the rapid screening of plant odors in culture.

Among the 150 components identified, a potent antifungal compound, 4-Ethylphenol, requires further exploration as a source or template for novel crop protection chemistry. There have been a few reports demonstrating the antimicrobial potential of 4-Ethylphenol (Xing et al., 2018). In this study, 4-Ethylphenol had a good inhibitory effect on *P. sojae* and *P. nicotianae*. Zoospore germination and mycelium growth are important for disease epidemics (Tyler et al., 1996). Our results showed that 1 mM (144.15 mg/L) 4-Ethylphenol completely inhibited *Phytophthora* spp. sporangium formation and mycelium growth. This inhibition is comparable to that of the plant-derived antifungal agent poacic acid when applied with an IC50 of 1,000 mg/L against *P. sojae* (Piotrowski et al., 2015). 4-Ethylphenol has a potent antifungal activity against three soil-borne phytopathogenic fungi, *Rhizoctonia solani*, *Fusarium graminearum*, and *Gaeumannomyces graminis* var *tritici*, and four *Fusarium oxysporum* forma specialis. Usually, these fungi are associated with *Phytophthora* spp. in the soil and cause compound infection complications, which aggravate the occurrence of plant root rot (Pemberton et al., 1998; Wen et al., 2017; Brown et al., 2021). The potent antifungal activity of 4-Ethylphenol could block the spread of these pathogens, and may play a key role in inhibiting soil-borne disease epidemics. This study provides the first report of the activity of 4-Ethylphenol against a series of *Phytophthora* spp. and fungi pathogens, demonstrates its potential as a universal broad-spectrum fungicide for soils, and justifies efforts to investigate its mechanism of action in detail.

To understand the mechanisms behind the antimicrobial action of 4-Ethylphenol, we examined the morphology of *Phytophthora* spp. mycelia treated with the VOC. Mycelium morphology could be changed after treatments with plant essential oils (Huang et al., 2019). Microscopic observation showed that *P. sojae* and *P. nicotianae* mycelia morphology was also changed after 4-Ethylphenol treatment. The leakage of intracellular materials, such as DNA and proteins, was significantly higher in treated mycelia than in the control, confirming that 4-Ethylphenol damaged the cell structure and thus affected the normal mycelial growth.

Because it is a potential antifungal agent, plant security studies for 4-Ethylphenol application are necessary. The application of 4-Ethylphenol in a certain concentration range (0–25 mg a.i./plant) did not inhibit or harm normal soybean and tobacco growth. At the lower but effective concentration range (5–15 mg a.i./plant), it even slightly promoted soybean and tobacco growth.

We further verified that 4-Ethylphenol is effective in controlling soybean root rot and tobacco black shank in pot experiments where, to mimic the field application, 4-Ethylphenol was mixed with soil. The results showed that 4-Ethylphenol could effectively inhibit both the soil-borne pathogens without affecting the normal plant growth. Future work should explore more effective application methods and lay a foundation for creating 4-Ethylphenol field application directives.

Botanical fungicides are derived from natural products and are less likely to develop drug resistance than chemical fungicides (Shuping and Eloff, 2017). None of the natural and eco-friendly chemical alternatives currently registered and available have the full spectrum of activity and versatility of methyl bromide as pre-plant soil fumigants (Duniway, 2002; Driver et al., 2016). Based on the results described here, 4-Ethylphenol is a potent antimicrobial that regulates plant growth and has the potential to substitute traditional antifungal agents.

## Data Availability Statement

The original contributions presented in the study are included in the article/Supplementary Material, further inquiries can be directed to the corresponding author/s.

## Author Contributions

QW and QX designed the experiments. TG, CL, and WG performed the experiments and analyzed the data. YW performed the GC–MS experiments and analyzed the data. TG and QW wrote the manuscript. QX and CH participated in manuscript revision or experiments. QW revised the manuscript and provided the funding for this research. All authors contributed to the article and approved the submitted version.

## Conflict of Interest

YW was employed by company Shimadzu (China) Co., Ltd. The remaining authors declare that the research was conducted in the absence of any commercial or financial relationships that could be construed as a potential conflict of interest.

## Publisher’s Note

All claims expressed in this article are solely those of the authors and do not necessarily represent those of their affiliated organizations, or those of the publisher, the editors and the reviewers. Any product that may be evaluated in this article, or claim that may be made by its manufacturer, is not guaranteed or endorsed by the publisher.
