# Supplementary material for: 4-Ethylphenol, A Volatile Organic Compound Produced by Disease-Resistant Soybean, Is a Potential Botanical Agrochemical Against Oomycetes
Source: Front Plant Sci. 2021 Sep 22;12:717258. doi: 10.3389/fpls.2021.717258 (PMC8492902; doi:10.3389/fpls.2021.717258)
Supplement: Supplementary file 1 [file Presentation_1.PPTX]

## Slide 1
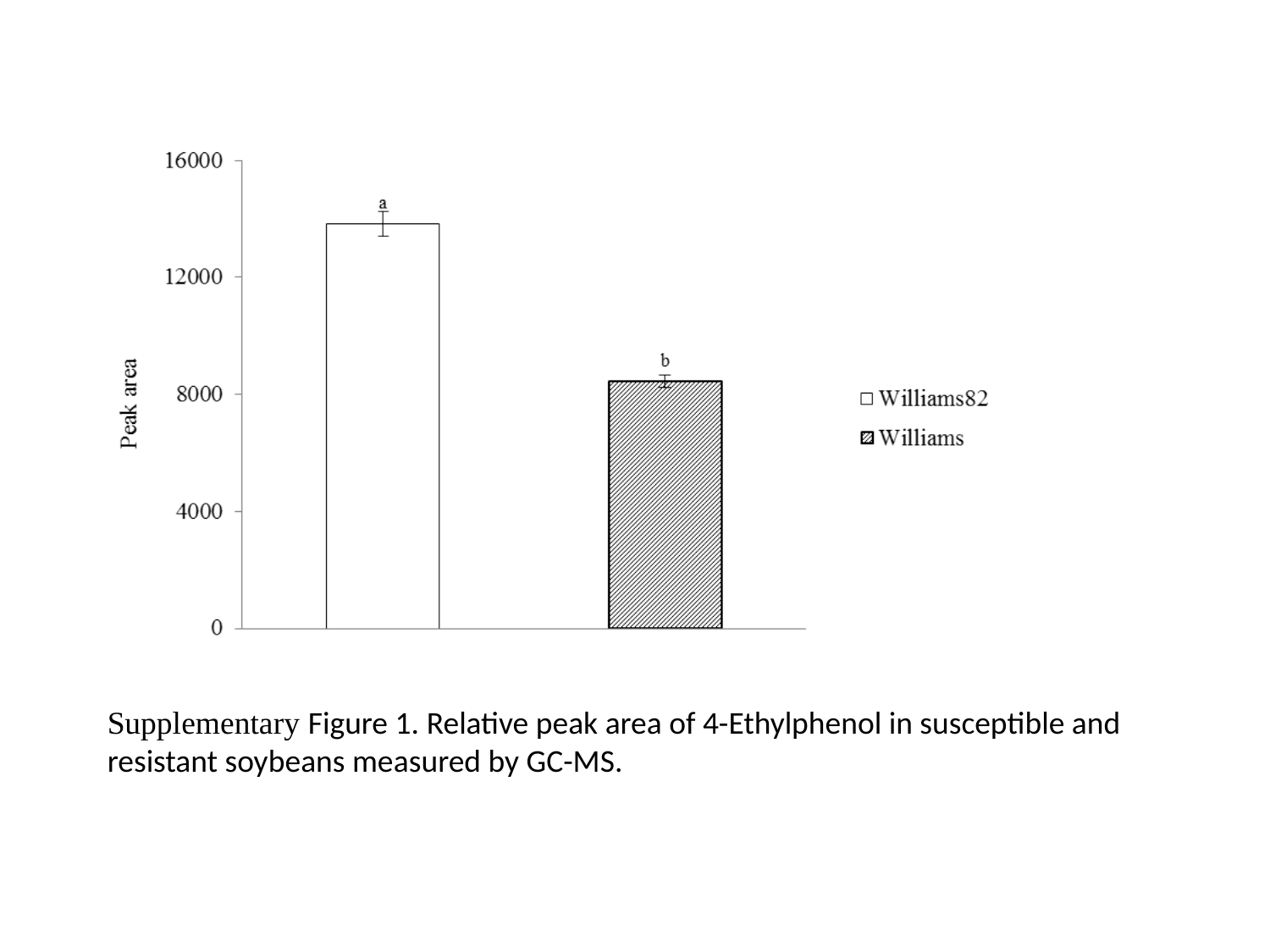

Supplementary Figure 1. Relative peak area of 4-Ethylphenol in susceptible and resistant soybeans measured by GC-MS.

## Slide 2
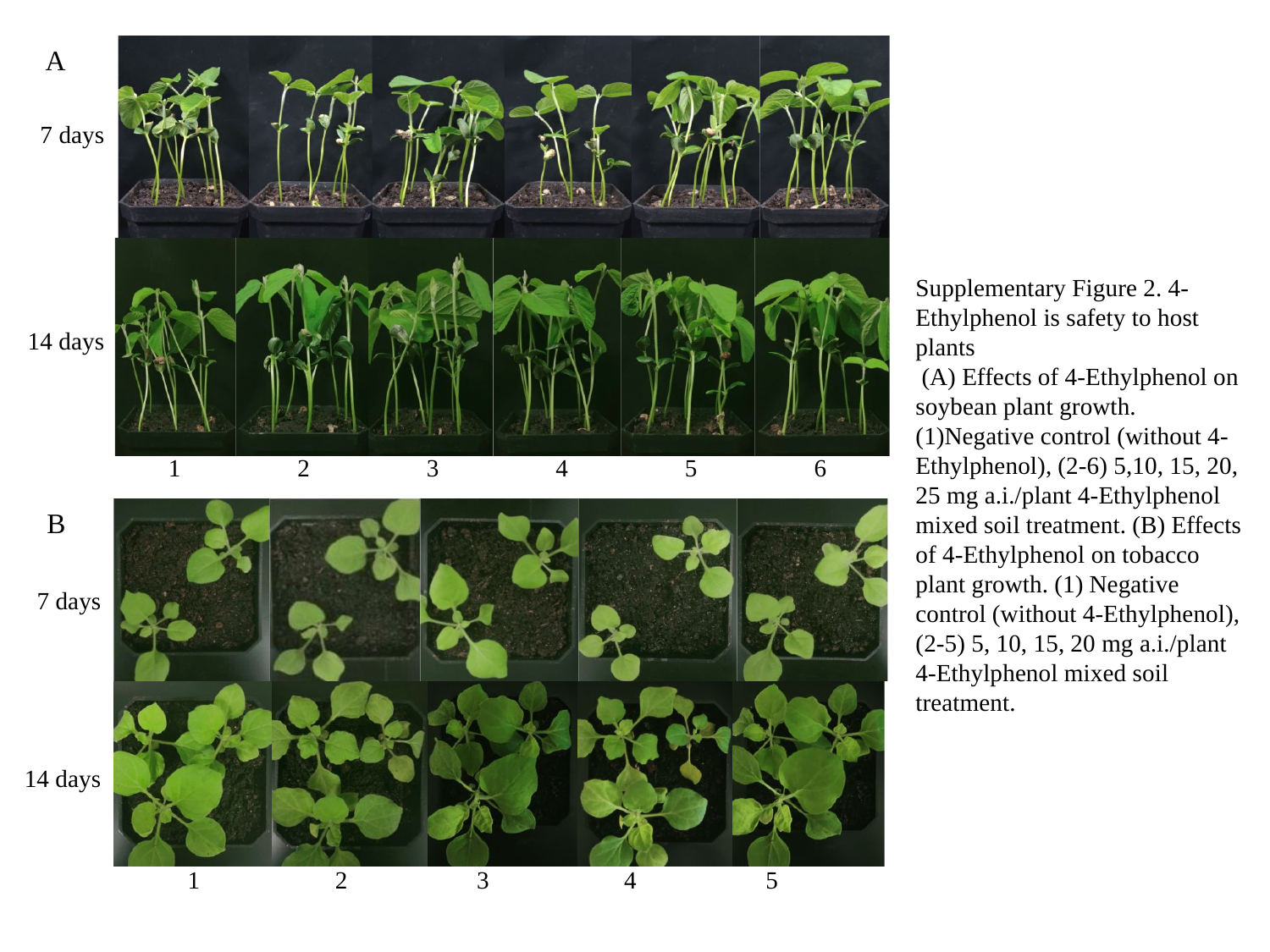

1 2 3 4 5 6
7 days
14 days
A
 1 2 3 4 5
7 days
14 days
B
Supplementary Figure 2. 4-Ethylphenol is safety to host plants
 (A) Effects of 4-Ethylphenol on soybean plant growth. (1)Negative control (without 4-Ethylphenol), (2-6) 5,10, 15, 20, 25 mg a.i./plant 4-Ethylphenol mixed soil treatment. (B) Effects of 4-Ethylphenol on tobacco plant growth. (1) Negative control (without 4-Ethylphenol), (2-5) 5, 10, 15, 20 mg a.i./plant 4-Ethylphenol mixed soil treatment.

## Slide 3
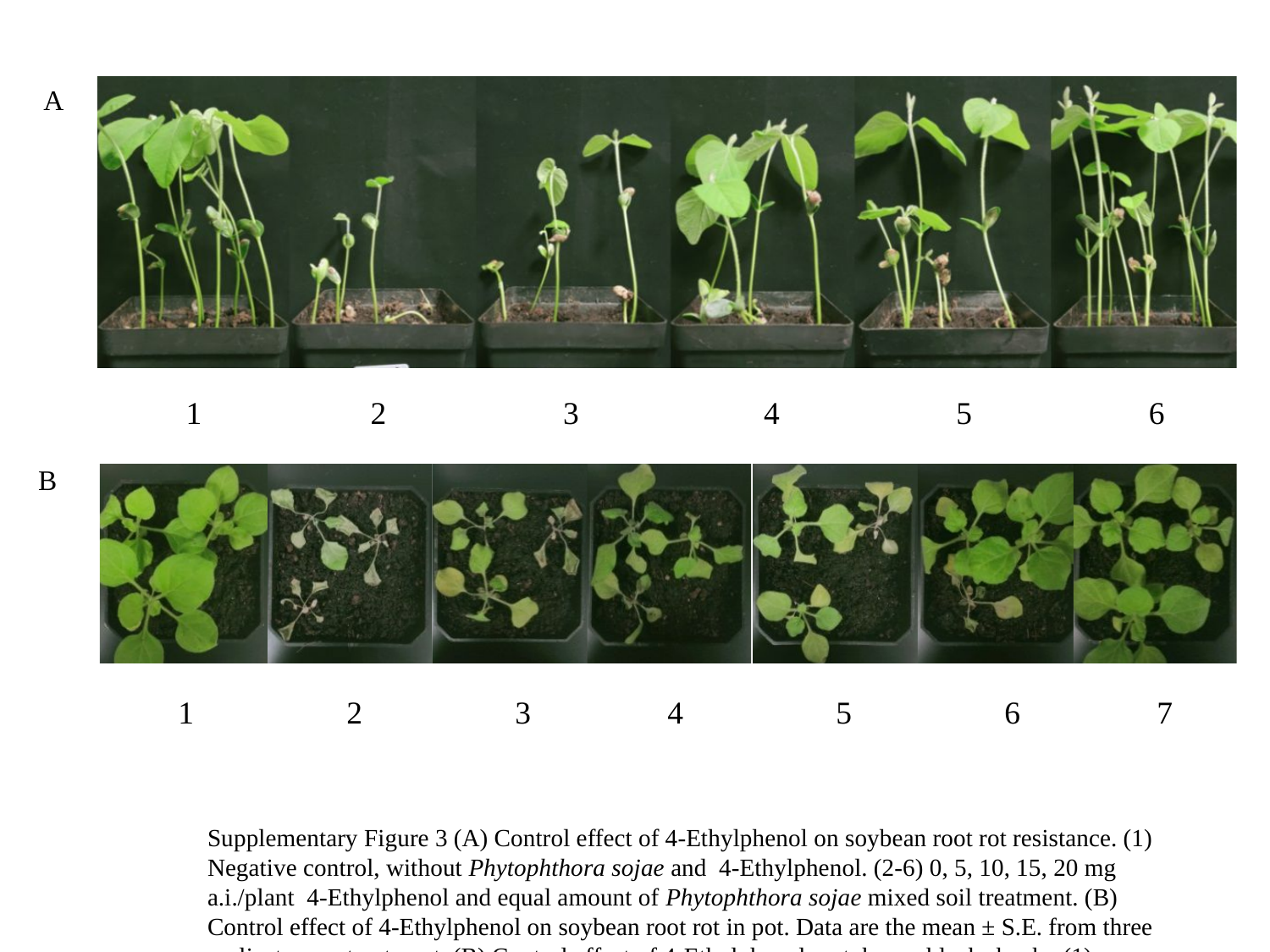

A
 1 2 3 4 5 6
B
 1 2 3 4 5 6 7
Supplementary Figure 3 (A) Control effect of 4-Ethylphenol on soybean root rot resistance. (1) Negative control, without Phytophthora sojae and 4-Ethylphenol. (2-6) 0, 5, 10, 15, 20 mg a.i./plant 4-Ethylphenol and equal amount of Phytophthora sojae mixed soil treatment. (B) Control effect of 4-Ethylphenol on soybean root rot in pot. Data are the mean ± S.E. from three replicates per treatment. (B) Control effect of 4-Ethylphenol on tobacco black shank. (1) Negative control, without Phytophthora nicotianae and 4-Ethylphenol. (2-7) 0, 5, 10, 15, 20, 25 mg a.i./plant 4-Ethylphenol and equal amount of Phytophthora nicotianae mixed soil treatment.

## Slide 4
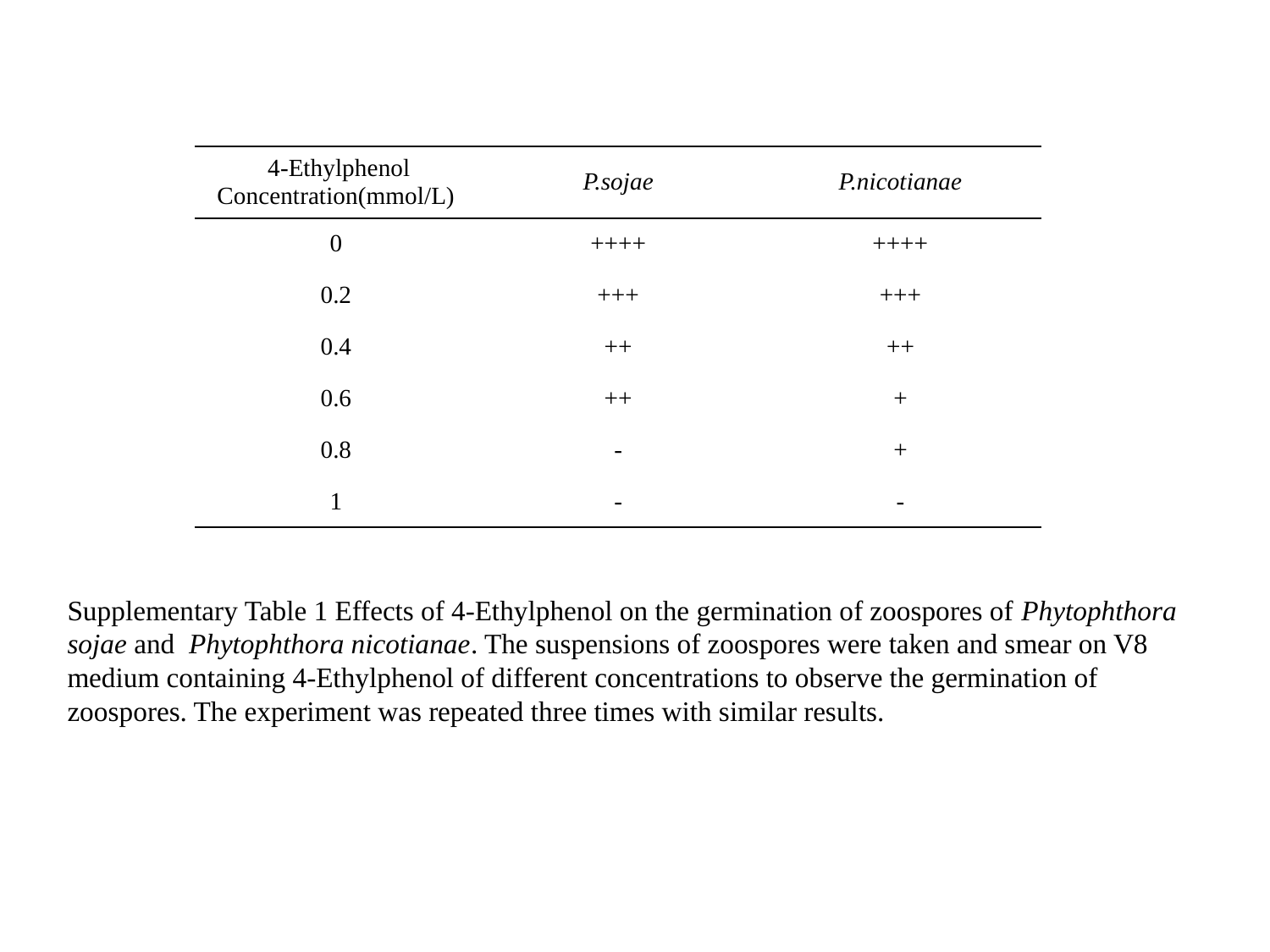

| 4-Ethylphenol Concentration(mmol/L) | P.sojae | P.nicotianae |
| --- | --- | --- |
| 0 | ++++ | ++++ |
| 0.2 | +++ | +++ |
| 0.4 | ++ | ++ |
| 0.6 | ++ | + |
| 0.8 | - | + |
| 1 | - | - |
Supplementary Table 1 Effects of 4-Ethylphenol on the germination of zoospores of Phytophthora sojae and Phytophthora nicotianae. The suspensions of zoospores were taken and smear on V8 medium containing 4-Ethylphenol of different concentrations to observe the germination of zoospores. The experiment was repeated three times with similar results.
